# Objective Fidelity Evaluation in Multisensory Virtual Environments: Auditory Cue Fidelity in Flight Simulation

**DOI:** 10.1371/journal.pone.0044381

**Published:** 2012-09-05

**Authors:** Georg F. Meyer, Li Ting Wong, Emma Timson, Philip Perfect, Mark D. White

**Affiliations:** 1 Department of Experimental Psychology, Liverpool University, Liverpool, United Kingdom; 2 School of Engineering, Liverpool University, Liverpool, United Kingdom; Bielefeld University, Germany

## Abstract

We argue that objective fidelity evaluation of virtual environments, such as flight simulation, should be human-performance-centred and task-specific rather than measure the match between simulation and physical reality. We show how principled experimental paradigms and behavioural models to quantify human performance in simulated environments that have emerged from research in multisensory perception provide a framework for the objective evaluation of the contribution of individual cues to human performance measures of fidelity. We present three examples in a flight simulation environment as a case study: Experiment 1: Detection and categorisation of auditory and kinematic motion cues; Experiment 2: Performance evaluation in a target-tracking task; Experiment 3: Transferrable learning of auditory motion cues. We show how the contribution of individual cues to human performance can be robustly evaluated for each task and that the contribution is highly task dependent. The same auditory cues that can be discriminated and are optimally integrated in experiment 1, do not contribute to target-tracking performance in an in-flight refuelling simulation without training, experiment 2. In experiment 3, however, we demonstrate that the auditory cue leads to significant, transferrable, performance improvements with training. We conclude that objective fidelity evaluation requires a task-specific analysis of the contribution of individual cues.

## Introduction

Synthetic multisensory environments, such as virtual reality systems or flight simulators are increasingly used for training in a variety of specialisations [1], [2] and there is evidence that sufficient realism is necessary for learning and transfer of new skills from simulator to reality [3], [4], [5], [6], [7]. There is therefore considerable pressure to implement high-fidelity simulations. Physical, computational and financial constraints, however, limit the fidelity that can be achieved and the sensory modalities that can be represented. The aim of this work is to propose a framework to evaluate the contribution of individual cues to overall human behavioural performance as a measure of fidelity.

### Measuring Fidelity

Fidelity is a term that is very commonly used and relatively easy to define as *a measure of the degree to which a simulation system represents a real-world system*. It is, however, much more difficult to operationalise the concept for objective evaluation. Schricker and co-workers ([8] p. 109), make no bones about their view that the ‘[…] main problems with how fidelity has been addressed […] are: 1.) No detailed definition; 2.) Rampant subjectivity; 3.) No method of quantifying the assessing of fidelity, and 4.) No detailed example of a referent […]’. They propose a fidelity evaluation framework that has three main features: An explicit definition of the relationship between the simulation and real-world system via a referent; a set of targeted comparisons between referent and simulation; and an explicit consideration of the application of the system.

The major contribution, in our view, is the acknowledgement that objective fidelity evaluation requires a ‘referent’, an abstract description of the real world that provides a definition of reality in a level of detail and format that makes a meaningful evaluation possible. The emphasis on application-specific and targeted comparisons between the simulation and referent reflects the view that the factors contributing to fidelity depend on the task, and that fidelity analysis aims to identify simulation components and behaviours that contribute to the overall performance of a simulation. In the context of visual fidelity Ferwerda [9] makes a distinction between physical (veridical stimulation of the sensory system), photo-realism (veridical representation) and functional fidelity (veridical representation of the ‘information’) in a visual scene and make the point that it is the functional specification of a scene that is particularly task relevant.

Jones et al. [10] in a review of simulation technologies highlight that physical correspondence is overemphasized as a fidelity measure for training purposes and argue that concern with fidelity should focus on achieving greater effectiveness and efficiency in terms of behavioural objectives. The view is mirrored more recently by Dahlstrom et al. [11] who showed there is no direct link between competence development and the realism of rendered scenes in simulation. They argue that lower-fidelity simulation, when appropriately designed, can provide competence development with pedagogical and economic advantages.

Standards do exist for the qualification of flight simulator training devices [12]. These standards typically detail the criteria for the cueing environment and the flight models and the training credits attainable for different levels of synthetic training devices. However, research undertaken by the GARTEUR (Group for Aeronautical Research and Technology in Europe) HC-AG 12 Action Group has questioned the validity of these general tolerances and has shown that the assessment of the fidelity of the device is sensitive to the type and duration of the task flown [13], which is consistent with Schricker’s argument. This led to work that actively seeks to include human behavioural data into simulator fidelity assessment methodologies [14] and metric based frameworks for the assessment of the fitness-for-purpose of a flight simulator [15], [16].

Within the fidelity evaluation framework proposed by Schricker et al. [8] we argue that the purpose of flight simulation is to provide human observers with signals that can be detected and discriminated, that either contribute directly to task performance, or that are the basis for transferrable learning. A key argument that we make, consistent with Jones et al. [10] and Dahlstrom et al. [11], is that the *referent* for fidelity evaluation is *human perception and performance* rather than descriptors of the physical environment. If greater realism, additional cues or simulation behaviours improve human performance, then this improves our operational definition of fidelity.

We argue that basic methodology from multisensory research can provide a robust and principled framework for evaluation of the contribution of individual cues or behaviours to fidelity.

The main advantage of using human behaviour as a referent is that quantifiable measures that show the *relative* contribution of specific cues to performance and training outcomes can be defined and experimentally obtained. These measures can directly contribute to design decisions, such as what cues to present or which behaviours to implement.

### Objective Evaluation of Multisensory Perception

Much of the information in real environments is represented in multiple modalities. Pilots, for instance, use aircraft motion to follow their predetermined flightpath [17]. This cue is directly represented in the visual domain and in kinematic cues that drive vestibular and tactile representations [18]. In addition to visual and kinematic motion cues, signals such as engine sound or wind noise provide important indirect information that pilots use [19], [20]. The relative contributions of different cues will vary from task to task and are dynamically re-weighted [21]: Visual cues, for instance, will provide strong vertical motion cues at and near ground level, but very limited information once an aircraft is at high altitudes.

Recent work on the psychology of multisensory perception has greatly advanced our understanding of how humans integrate cues from multiple modalities (for a review see [22]). With this development came efficient methods to evaluate human performance in multisensory environments and formal models that describe how cues are integrated and how individual cues contribute to overall behavioural performance. These methods and models have been applied in areas such as automotive interface design [23] and flight simulation [24], [25], [26].

Our aim is to show how multisensory perception measures can form the basis for a fidelity evaluation framework that uses human performance as a referent and is designed to evaluate the relative contribution that individual cues or behaviours make to simulated environments.

We present data from three experiments to show how the contribution of cues in multisensory environments can be objectively measured. All experiments use the same flight simulation environment but explore different tasks and performance metrics (see methods section for details). We concentrate on the contribution of auditory cues to a helicopter flight simulation but the fidelity evaluation methodologies can be applied much more generally.

The first requirement for any cue in a simulated environment is for it to be sufficiently salient to be reliably detected. Where a cue carries semantic information it must be correctly categorised. In experiment 1 we measure thresholds for the detection and categorisation of auditory and kinematic signals that cue helicopter motion in the simulated environment. We show that both cues are detectable in the flight simulator and that the simultaneous presentation of the two redundant cues increases detection performance significantly beyond the level seen for single cues. This is a hallmark of multisensory integration and a useful fidelity measure because the effect is typically only seen if the two signals are well matched [27], [28], [29].

In many situations multiple, non-redundant, cues contribute to our performance. Visual motion cues, for instance, are normally disambiguated by somatosensory and vestibular information that enables us to discount self motion from the visual signal (e.g.[30]). In a second experiment we measure target-tracking performance while systematically manipulating the auditory and kinematic cues available to the participants. This experiment shows that our participants make effective use of kinematic, but not auditory cues to improve their behavioural performance. We hypothesize that this is not due to a lack of salience - we demonstrated that the audio cues are correctly perceived in experiment one - but because participants have to learn the complex mapping from the turbine noise to aircraft movement to carry out the tracking task.

In the third experiment we investigate whether participants can learn to use this auditory cue during normal operation in a simulated environment. We employ an implicit learning strategy where participants are exposed to informative audio signals but not explicitly instructed to attend or use the signals. In analogy to the transition between real aircraft and flight simulators, which offer a much reduced fidelity, our participants are tested in a flight simulator with high fidelity graphics and a motion platform but trained in a reduced fidelity environment without motion cues and with limited visuals. We show that target-tracking performance of our participants rapidly improves during training and that implicitly learned audio cues improve performance in a final test in the full simulator.

### Experiment 1: Evaluating Cue Fidelity by Detection and Categorisation Performance

Our sensory systems do not work independently, but integrate information from many modalities to ‘make sense’ of our environment. Signals that represent temporally, spatially, and semantically congruent information are detected or discriminated faster or more accurately than incongruent bimodal stimuli (for reviews, see [28], [29], [31]). The facilitatory effect of spatial and temporal congruence can be explained by early neural integration stages that have, for instance, been demonstrated in the superior colliculus of cat (e.g. [32], [33]). Semantic congruency effects are more likely to be mediated by high-level cortical mechanisms because of the required categorization of the underlying stimuli into meaningful signals (e.g. [34]).

A basic requirement for any simulation is that typical signal changes are detectable and that signals representing different semantic categories, up and down motion in our example, can be categorised correctly. Signal detection tasks provide an efficient and robust method to evaluate the relative contribution of the cues that drive our perception and performance in simulated environments. Formal models of multisensory integration make strong predictions about human performance in situations where information is represented in multiple modalities: Congruent information should have a facilitatory effect which is an important fidelity indicator [28], [35].

We report data on the detection and integration of auditory and kinematic motion signals in a flight simulation setting. Participants were required to report upward or downward changes that were cued either via the motion platform, changes to the sound of the simulated helicopter turbine, or both. Changes were reported by pushing the top-hat button on the cyclic control stick in the flight simulator either up or down. We employed a forced choice paradigm, which required participants to answer after each visually cued trial. If no signal changes are perceived, or if they cannot be categorised, participants will perform at chance level (50% correct identification).

We tested at five levels of control input changes (Xc = +− 0.1, 0.2, 0.3, 0.4, and 0.5), which corresponds to collective [footnote reference 1] movements between 0.1 and 0.5 inches in conditions where pilots control the aircraft, see methods section, below, for more details. We measured a *mean* absolute displacement of 0.91 inches (s.d. = 0.81) with equivalent flight dynamics in experiment 2 where our participants controlled the flight. Detailed descriptions of the participants, the flight simulator and stimuli used are given in the methods section at the end of this paper.

[Footnote 1: The collective pitch control, or *collective*, is a lever on the left of the pilot seat that controls the pitch angle of the main rotor blades and therefore the lift. Increasing the pitch angle for more lift requires more engine power, which causes the turbine sound to increase in pitch and amplitude. The main rotor speed is kept approximately constant in normal flight conditions.].

**Figure 1:**
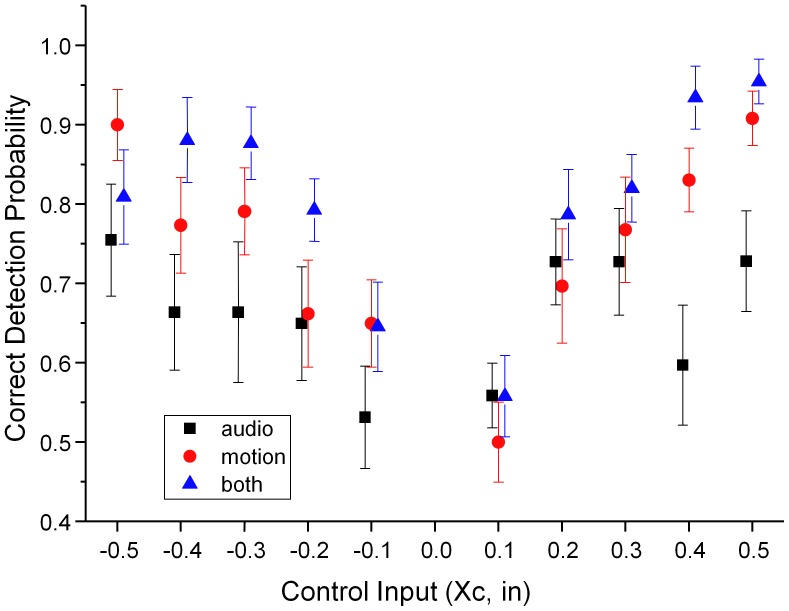
Raw cue detection performance at control input levels ranging from −0.5 (down) to +0.5 (up). Subjects were required to respond at all trials so that chance performance is 0.5 (50% correct). Error bars are standard error of the mean (SEM). The data points are slightly offset to enhance visibility.

The data for 10 participants, unfamiliar with the flight simulator, shows that in each of the three conditions correct categorisation rates increase with cue magnitude, fig 1.

Paired t-tests comparing the correct detection rates across the three conditions show that all three conditions result in significantly different performance rates (audio vs motion: t_(109)_ = −3.76, p = 0.00027; audio vs both: t_(109)_ = 6.17, p<0.0001; motion vs both: t_(109)_ = −3.43, p = 0.00084).

Paired t-tests comparing each of the equivalent motion conditions in the up/down direction revealed no significant differences (audio t_(54)_ = −0.55, p = 0.60; motion t_(54)_ = 0.39, p = 0.71; both t_(54)_ = −0.23, p = 0.82) so that for further analysis equivalent up and down motion conditions are pooled.

**Figure 2:**
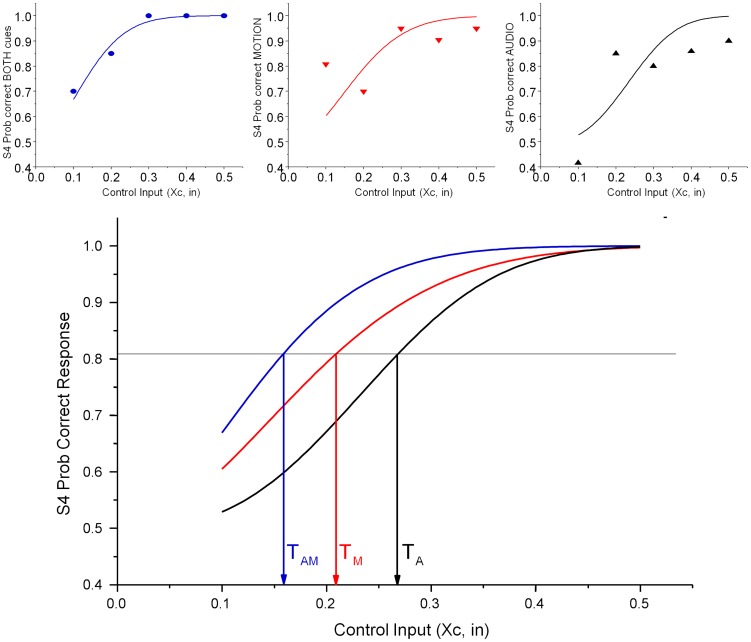
Sample experimental data (top) and curve fits for one subject (S4). A sigmoidal Weibull function was fitted to each individual data set. The threshold is defined as the point where participants correctly identify motion in 81% of trials. In this case the motion thresholds are (Audio: T_A_ = 0.27; Motion T_M_ = 0.21; Simultaneous presentation for both cues T_AM_ = 0.16).

One of the key contributions from behavioural studies of multisensory integration is that congruent, redundant signals, such as auditory and visual motion cues facilitate detection (e.g. [28], [31]). Formal models enable us to differentiate whether two signals are integrated at very early processing stages in the brain (linear summation, e.g [36]), whether they are integrated in a statistical (optimal) sense [35], [37], or whether they are processed independently. A key measure is the detection threshold. This is established from the psychometric function (fig. 2) which relates detection or correct classification probability against signal strength.

A well established method (e.g. [38] [36] [39]) is to fit a sigmoidal function and to define the inflection point as the threshold. We fitted a Weibull function [37]:
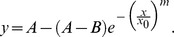



Parameters A and B define the lower and upper asymptote of the function and were fixed at chance performance (p = 0.5) and maximal performance (p = 1.0) respectively. The parameter *x_0_* describes the level at which 81% of signals are correctly detected, while the slope of the curve, an indication of the decision reliability, is described by parameter m. Figure 2 shows an example fit to the experimental data for one participant. Each data point represents the mean probability that a control signal (Xc, see methods section) of a given absolute strength is correctly identified.

**Figure 3:**
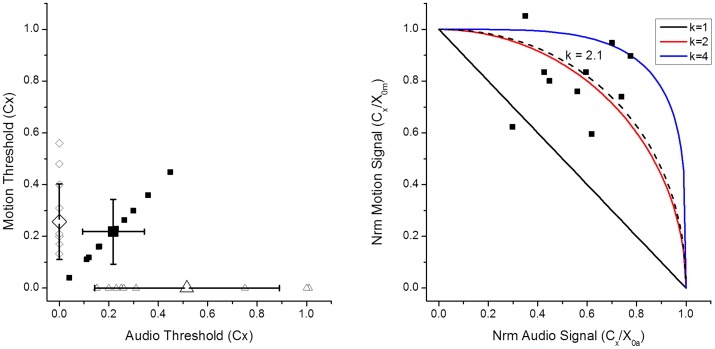
Threshold data for individual subjects and means thresholds (left). The Quick pooling model quantifies integration by fitting a line to the normalised threshold for each subject (right). For our data the best fit is achieved with a pooling factor of k = 2.1. A linear summation model, consistent with early sensory integration would predict a pooling factor of 1 (black line).

The threshold estimates for all subjects together with overall mean thresholds are shown in figure 3 (left panel).

The data shows that changes to motion and auditory cues are perceived and correctly categorised by our participants, and that simultaneous changes to both cues lead to a reduction of the overall detection threshold (and response variance) compared to unimodal signal changes. This is evidence for effective integration of both stimuli.

Quick (1974) proposed a relatively simple metric to evaluate how cues are combined when more than one cue is present relative to situations where single cues are presented. The bimodal thresholds are replotted in a space defined by unimodal thresholds (threshold units, 1) for each observer. The distance of the threshold from the origin, when multiple rather than single cues are presented can be evaluated using the Minkowski distance metric: 
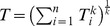
 where *T* is the threshold that is the result of presenting *n* cues, each with individual thresholds *T_i_*. A pooling factor, *k*, defines the distance of all individual thresholds from the origin. A pooling factor *k = 1* is seen when the signals representing both underlying cues are linearly combined before decisions are made, diagonal line in figure 3 (right panel). This is usually referred to as a linear summation model. Pooling factors of around *k = 4* are typical of probability summation models where individual cues are evaluated and local decisions combined [36]. Optimal Bayesian integration, where cues are processed and decisions are made individually, but where the relative contribution of each cue is weighted by its reliability results in a Euclidean distance metric (*k = 2*, [40]). In cases where individual cues are not combined at all, but where the joint threshold is crossed whenever one underlying cue is detected, *k* tends to infinity.

In our example with two cues (audio *a* and kinematic motion *m*) the combined threshold is given by 
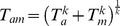
.

Fitting the model to our observed threshold data results in a pooling factor of *k* = 2.1, (dashed line in fig. 3, right panel) which suggests that the two cues are optimally statistically integrated.

Our experimental results clearly show that the auditory and the kinematic motion cues can be detected and are correctly associated with up/down motion. Presenting both cues simultaneously has a facilitatory effect by reducing the threshold consistent with a model that assumes optimal integration of both cues.

For a task where cue detection or categorisation are key requirements, or where transferrable learning depends on the correct processing of these cues, we demonstrate that auditory *and* kinematic motion cues make a clear contribution to fidelity. Signal detection provides an efficient and robust method to evaluate whether individual cues contribute to human perception and therefore provide a key first stage in a human-centred, cue and task specific fidelity evaluation framework.

### Experiment 2: Evaluating Cue Fidelity by Performance Measures

Dynamic systems, whether they are our own bodies or flight simulators, can be modelled by transfer functions that translate a control input into complex behaviour. These models form the basis for the prediction of the consequences of a control input to behaviour which is an essential aspect that enables us to operate in novel or changing environments.

In motor behaviour, the transformation from motor commands to their sensory consequences is governed by a complex interplay of the environmental factors, the musculoskeletal system and sensory receptors (review [41]). Cues from multiple modalities, such as vision, haptics and vestibular information are combined to build predictive models of behaviour where each modality provides different, complementary signals to build novel representations (review [42]).

This is not unlike situations where humans operate machinery, where control inputs are also mapped into complex behaviours, which can be predicted from a range of complementary cues. The heave (vertical motion) model, used to model flying height in flight simulation, is used as an example in the following experiment. The simulation provides a rich set of sensory signals that represent different stages of the transfer function: Haptic (collective position) and auditory signals (turbine noise) provide a direct representation of the control input to the heave model. Acceleration is cued via the motion platform, providing somatosensory and vestibular signals, while, at the end of the control model, flying height is represented visually. The simulation also includes an explicit auditory error signal that represents the difference between the visual target and actual position.

Participants were asked to follow the height of a visually presented target, a refuelling basket, for relatively short (2 min) periods in a helicopter refuelling simulation. The heave model damping parameter (Z_w_, see methods), and with it the flight characteristics of the aircraft, changed in a pseudorandom sequence. Our 10 participants consequently had to rapidly discover and use the system transfer function to minimise the error between the target and real flying height.

The vertical target motion seen by the ‘pilot’ is a compound of target and self-motion, so that cues representing self-motion, such as the turbine noise and kinematic cues can make a major contribution in disambiguating the visual signal.

**Figure 4:**
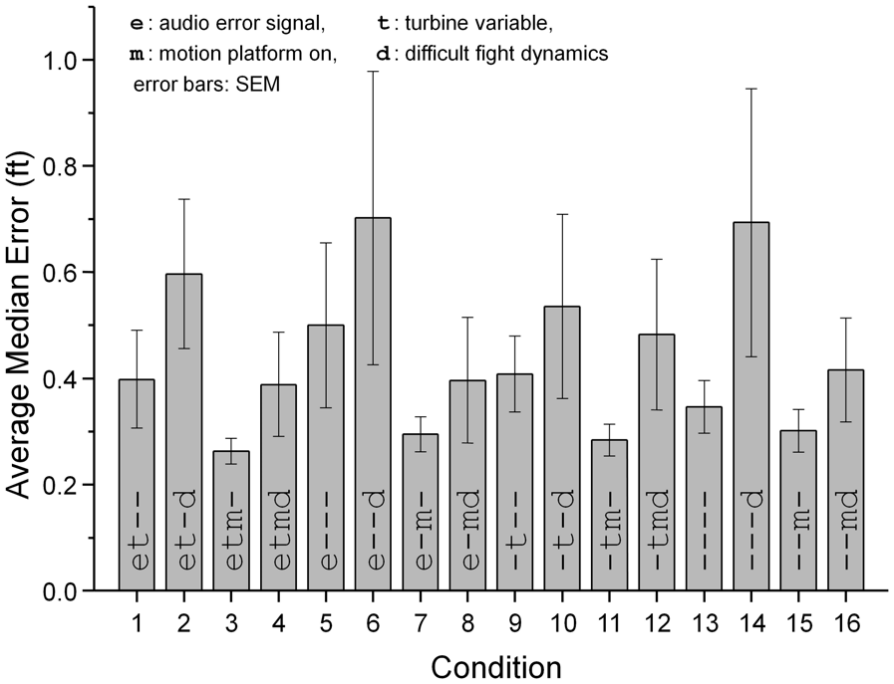
Average median error over 10 subjects for each of the 16 test conditions. A factorial design that combined each of the four factors: Audio error signal off (-)/on(e), turbine sound static (−)/variable (t), motion platform off(−)/on(m) and flight dynamics difficult (d) or easy (−) were used.

To evaluate the relative contribution of auditory and kinematic cues, the experiment was run as a factorial design where four factors were systematically explored: The motion platform could be on or off (labelled *m* in fig 4), the auditory turbine simulation could signal the control input (*t*, fig. 4) or produce a static sound and the distance to the target (error, *e*) could be signalled by an auditory beep or not. Subjects were tested using two flight dynamics models, difficult (*d*, fig. 4) or easy. Each of the subjects was tested in a pseudorandom sequence of all 16 test conditions that resulted from 2×2×2×2 possible cue combinations. The order of testing was balanced to exclude learning effects.

Human performance was measured as the median absolute distance (error) between the refuelling basket and the aircraft altitude. The mean error over all subjects is shown in figure 4. Visual inspection shows that the error is consistently larger in the difficult flight dynamics conditions compared with the easy conditions (difficult: *d*, fig. 4 - even vs odd numbered conditions). On average, errors are also smaller in conditions where the motion platform is on (labelled *m*, fig. 4 - conditions 3&4 vs 1&2, 7&8 vs 5&6 etc) compared to conditions where no platform motion is present.

**Figure 5:**
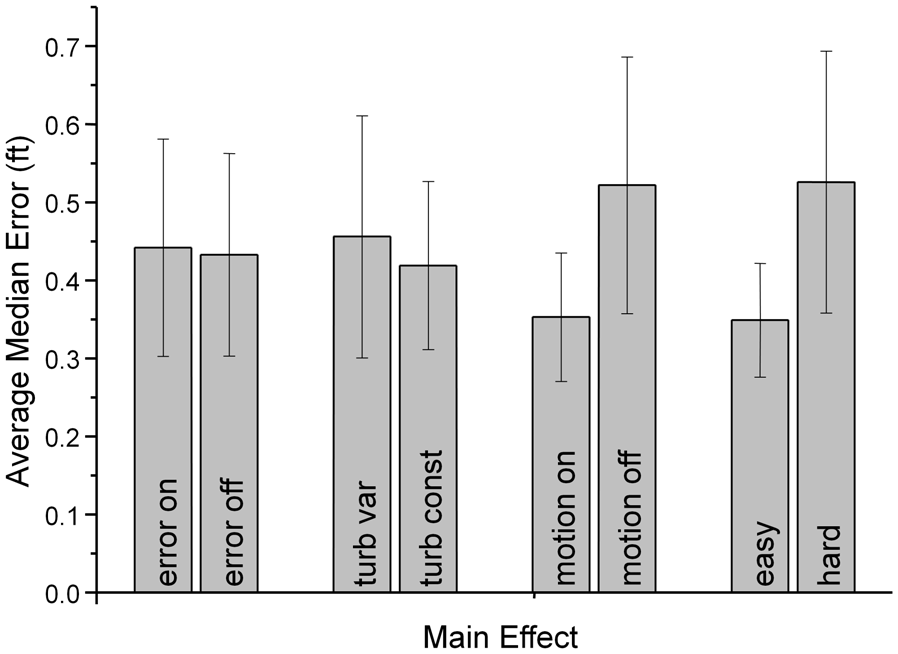
Main effects for the target-tracking experiment. The average median error is plotted for the eight principal experimental conditions. Neither an explicit error signal (error on/off), nor turbine sound modulation (variable vs. constant turbine sound), affects target-tracking performance. Kinematic cues from the motion platform, however, significantly improve performance. Subjects also perform significantly better in the ‘easy’ flying condition overall.

The main advantage of the factorial design is that an analysis of variance (ANOVA) can be used for inferential statistics to evaluate the contribution that each of the factors (cues) makes to tracking performance. The main effect is the effect of a specific factor averaged over all other experimental conditions, fig. 5. This data pooling provides robust estimates of the contribution of each cue to overall performance and fidelity.

An ANOVA with subjects as a random factor shows significant main effects for the difficulty level (F(1,159) = 12.48, p = 0.006) and motion cues (F(1,159) = 11.44, p = 0.0009). Neither the turbine audio cue (F(1,159) = 0.054, p = 0.46), nor the error signal (F(1,159) = 0.03; p = 0.86) affect tracking performance, fig. 5. There were no significant interactions.

Our participants were untrained and we deliberately limited their ‘flying’ experience to 32 minutes in total to minimise learning effects. The results show that the heave model behaviour (easy/hard) makes a difference to overall performance, which was expected. Acceleration cues from the motion platform (motion on/off) made a highly significant difference to tracking performance, while neither of the two audio conditions had a significant main effect.

In experiment one, we showed that participants were able to detect and categorise the turbine signal and that the presence of the signal significantly enhanced performance in the bimodal condition compared to the motion condition alone. This experiment shows that successful detection and categorisation of our auditory signals is not sufficient for the tracking task, which requires a forward model of the aircraft behaviour. To predict the response of the heave model, it is not only the *presence* of a pitch/amplitude change that has to be detected, but this change has to be quantified and incorporated into a model of flight dynamics. We deliberately limited exposure to the flight model to prevent learning.

In this context the most striking result is not the failure to exploit auditory cues, but the finding that physical motion cues are immediately useful for the disambiguation of the visual signal. Physical motion cues are an integral part of our everyday, visual, experience and essential to estimate veridical motion from visual signals, in other words we are very well adapted to use kinematic cues to disambiguate visual motion signals (e.g. [42]).

The immediate accessibility of kinematic cues for our participants shows that the motion platform behaviour and signals match that seen in everyday environments and therefore is a useful diagnostic for objective fidelity evaluation.

The two audio cues, representing motion in our experiment, are not part of our everyday environment; therefore require learning to be useful.

### Experiment 3: Evaluating Cue Fidelity in Terms of Transferrable Learning Performance

The principal reason for using flight simulators is to provide transferrable and persistent training, objective measures of the contribution that specific subsystems make to fidelity and training outcome are important issues for the design and qualification of flight simulators.

The relative contribution to fidelity and training outcome of kinematic motion cues is a particularly hotly contested topic: There is no question that kinematic cues improve pilot acceptability and improve pilot performance, particularly for disturbance tasks, such as in turbulence [1] or in the tracking task described in experiment 2. Neither subjective acceptability of the simulation, nor performance in the simulator, however, provide evidence for transfer of training from the simulator to a real aircraft, in particular since the motion cues generated by many simulators fall short of those experienced in real planes. Bürki-Cohen and Sparko [17], for instance, argue that the success in training pilots in simulators with inadequate kinematic cues suggests that platform motion is not needed for a successful training outcome in fixed wing aircraft.

We are not going to resolve this debate but argue that it highlights two issues raised by Schricker et al. [8] for objective evaluation: One issue is that fidelity (and training outcome as an operational definition of fidelity) is highly task-specific, therefore any evaluation should take this into account. The second issue is that meaningful evaluation requires a referent, which real aircraft in most situations cannot provide. To evaluate the specific contribution that cues, such as platform motion or engine sound, make to the learning outcome, specific and targeted tests, such as those described in experiment two are necessary. We propose a framework that evaluates whether transferrable training for a specific task is aided by specific cues. This incremental approach enables us to use behavioural measures as reference data: We expect to see cue-specific performance improvements during training that are robust to changes in the environment and to changes to cues that are not task relevant.

In experiment 2 we showed that participants can use kinematic motion cues, but not auditory cues to disambiguate self-motion and target-motion and speculate that the indirect relationship between the simulated turbine noise and helicopter motion requires training to be useful. To show transferrable training we evaluate target-tracking performance with 10 participants that have not been exposed to a flight simulator using the same task and equipment.

In analogy to transferrable pilot training in a flight simulator that will be applied in real aircraft, we test our participants in a full flight simulator with a motion platform, a high quality collimated visual display and realistic control inceptors before and after training. The training, however, takes place in a much lower fidelity environment, our simulator-simulator (simsim). The visual representation is reduced to a schematic and there is no motion platform (see methods section), but the flight dynamics model and auditory signals are identical to those used in the Heliflight simulator [43]. Our participants were asked to use the collective lever to keep an ‘x’ on the screen within a larger circle, representing the refuelling basket used in the full simulator experiments. We used an implicit training paradigm: The turbine sound simulation was played throughout each training run, but instructions to the participants contained no reference to the sound.

**Figure 6:**
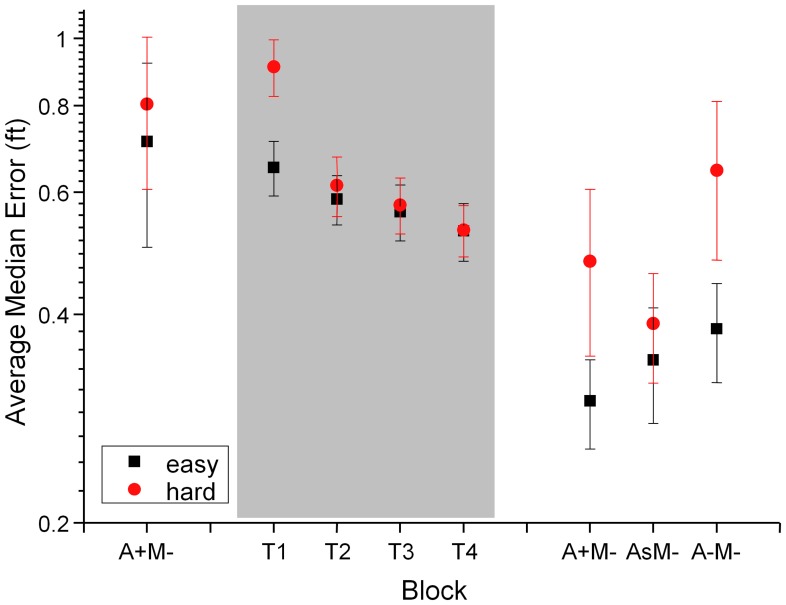
Target-tracking performance as mean error (ft) and SEM for ten participants before (left of grey box), during (grey box, T1–T4) and after training (right of grey box). The data shown represents easy (black) and hard (red) flying conditions. Test conditions are labelled as follows: T1–T4, 15 min training blocks, A+ : turbine signal amplitude and pitch modulated to represent the control input (x_c_), A- : turbine signal static, As : substitute sound in test conditions. The motion platform was off in all test conditions reported in the graph (M−). We see a significant reduction in tracking error during training (grey box) and between the initial (A+M−, left) and final test (A+M−, right) in the full simulator. After training performance in the condition without audio cues (A−M−) is significantly worse than when cues are present (A+M−). Substituting the turbine noise used during training for a different sound that exhibits the same behaviour (AsM−) does not significantly affect performance.

Figure 6 gives an overview of the results. Since training was carried out without a motion platform only results that are directly comparable are reported. The leftmost bar (A+M−) shows the average median error for the 10 participants in a two minute target-tracking experiment in the easy and difficult flying conditions preceding the training. The next four data points, underlaid in grey, show the mean error during four successive 15 min training sessions in the low fidelity environment (simsim). On the right of the data reporting training performance are three test conditions, which, like the initial test, were conducted in the HELIFLIGHT simulator. Condition (A+M−) is the same as during the initial test. A pairwise t-test comparing tracking performance for each subject in matching conditions before and after training shows a reduction in average tracking error from 0.74 ft (se = 0.19) to 0.39 ft (se = 0.091) ft (t = 6.76, df = 79, p<0.0001). This shows that training in the reduced fidelity simulator leads to significant transferrable target tracking performance improvements.

**Figure 7:**
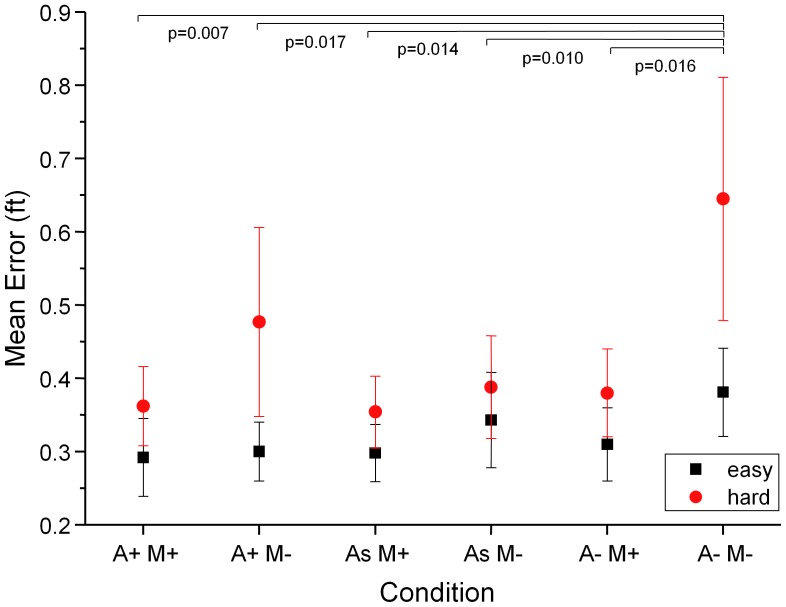
Target-tracking error after training. Error bars are SEM. We compare three audio conditions: turbine modulated (A+), turbine static (A−) and substitute sound (As), and two motion conditions: motion platform on (M+) and off (M−). Pairwise comparisons over both flying conditions (hard, red; easy, black) show significant differences between the condition where neither audio, nor motion cues (A−M−) help to disambiguate visual cues in the tracking task and the other conditions.

To test whether the participants learnt to use the audio cues and to what extent training depends on the precise nature of the audio signal we tested our participants with three different audio signals: The exact audio configuration that was used during training, a static turbine sound that did not provide meaningful audio cues (condition A−M−, fig. 6) and a substitute sound (condition AsM−, fig. 7), chosen to be obviously different from the turbine sound used during the training, but exhibiting exactly the same behaviour. We used a saxophone tuning note, which was amplitude and frequency modulated proportional to the control input Xc in exactly the same way as the turbine sound. If training depends on the physical characteristics of the acoustic carrier signal, then performance for the substitute sound should be no better than for the static sound. If participants use the cue modulation as a functional cue to fidelity [9] then the turbine sound and saxophone sound should both provide useful information.

After training we see much larger differences between the hard and easy flying conditions compared to the training conditions. One reason for this is that, during training, 15 minute blocks with constant flying conditions were used, so that participants had an opportunity to adjust fully. During initial and final testing, each block only lasted for 2 minutes. The full simulation also contained additional visual cues, the tanker plane at a fixed height, which may explain why participants performed better during the easy final tests than during equivalent easy training sessions.

A 2×2×2 ANOVA with the factors turbine (variable/static), motion (platform on/off) and difficulty (hard/easy) shows the expected significant main effects of difficulty: the mean tracking error reduces from 0.41 ft (se 0.10) in the hard conftion to 0.33 ft (se 0.08) in the easy condition (F(1,119) = 9.09, p = 0.0092), motion cues significantly reduce the error from 0.41 ft (se.11) to 0.34 ft (se 0.066) (F(1,119) = 5.98, p = 0.0015) while the mean error measures for the three audio conditions were 0.41 (se 0.11) for static audio, 0.37 (se 0.10) for variable audio cues and even lower at 0.33 (se 0.05) when the substitute sound was played (F(1,119) = 3.34, p = 0.034). No significant interactions were found. Subjects were coded as a random factor.Detailed performance data for all post-training test conditions are shown in figure 7. Post-hoc tests (one-sided paired t-tests to test the hypothesis that additional cues would reduce the error) over both flight dynamics conditions (p values are shown in figure 7) show that the mean error in the ‘no-motion, static-turbine’ condition is significantly larger than in any of the other conditions. The results show that audio and motion cues significantly contribute to performance after training. We see performance improvements if either the kinematic *or* auditory motion cues are present, presenting both cues together (condition A+M+) does not lead to significant performance increases compared to conditions where one cue is present (A−M+, A+M−). This finding contrasts with the results of experiment 1, which showed a significant detection performance enhancement when two cues rather than one are present. The performance measure in experiments 2 and 3 is more variable than the threshold estimates used in experiment 1, which may explain this finding. The result also highlights the need to use sensitive performance measures and robust statistical analysis techniques, such as within subject comparisons and factorial designs to maximise the sensitivity of the tests. Longer test runs or more subjects would also have reduced the variability inherent in behavioural tests, but an important consideration in the design of our experiments was to ensure that relatively sensitive measures of performance and fidelity are viable with limited testing.

## Discussion

Objective fidelity evaluation requires carefully defined metrics that enable a systematic comparison between the simulation and the system to be modelled. In many cases, such as flight simulation, objective descriptors of reality are not readily available, cannot be replicated in a simulation or do not contribute to the learning outcomes. It is, for example, not realistically feasible to obtain measurements and models of all aircraft behaviours under all flying conditions, such as the engine noises that we simulated in our experiments. Physical limitations of the simulator mean that many original cues cannot be represented faithfully. The most obvious example in flight simulation is aircraft motion which is constrained by the physical limitations of the motion platform so that washout filters are used to provide the pilots with the illusion that real motion takes place. Even if certain aspects of reality can be faithfully modelled, they may not be task-relevant and therefore do not contribute to learning outcomes: It is arguable that the fidelity of a simulated approach to a runway is not enhanced by modelling the behaviour of cows in an adjacent field.

A key argument we make is that the referent for any objective evaluation of fidelity should be human perception and performance rather than physical reality. Recent advances in multisensory perception show that human observers actively integrate sensory signals from multiple modalities to enhance their performance. This research provides us with experimental paradigms that enable us to obtain robust performance measures, and with formal models against which experimental data can be tested.

In experiment 1, we show that kinematic and auditory motion cues are not only detected independently but effectively integrated so that when both cues are present simultaneously human detection thresholds are consistent with predictions made by an optimal statistical integration model. Experiments that measure detection thresholds provide a robust framework to evaluate whether individual cues contribute to a simulation.

Temporal synchrony is one of the main determinants for effective audio-visual integration (e.g [44]) and an important determinant of simulator fidelity [45]. Simultaneity judgements show that the perception of galvanic vestibular stimulation lags behind vision by 120–160 ms [46]. When the timing of active and passive head movements relative to visual, auditory and tactile stimuli is manipulated, delays between 45 ms (passive head movements) and 80ms (active head movements) are necessary for the comparison stimuli to be perceived as simultaneous with head movements [47]. The kinematic signals in our flight simulator are delayed by approximately 80 ms relative to control input. The evaluative framework we propose would be very well suited to test whether a reduction in kinematic delays would lead to improved detection performance (experiment 1) or a reduction in tracking error (experiments 2/3). Reaction time measurements and formal models that predict response times for multimodal signals from single cues (e.g. [44]) would be an appropriate methodology.

We argue that fidelity evaluation should be task-specific: Our experiments show that the contribution of auditory and kinematic cues signalling aircraft motion depends on the specific task. Auditory cues can be detected and identified as signalling up or down motion without prior training. For auditory cues to contribute to performance in the refuelling basket tracking task, however, our participants required training (experiments 2 and 3).

The experimental paradigm we propose explicitly evaluates the contribution of individual cues or cue behaviours to human performance as an objective fidelity measure. This approach enables us to evaluate relative performance (and fidelity) changes that are introduced with additional cues and therefore can directly aid design decisions.

One of the primary application areas for simulation, in particular flight simulation, is training. It is essential that task relevant cues and behaviours that are part of the simulation contribute to transferrable learning. We used an implicit learning strategy to train our participants to use audio signals to disambiguate the visual signal in a reduced fidelity environment. We show that target-tracking performance of our participants rapidly improves during learning and that implicitly learned audio cues improve performance in a final test in the full simulator. Our data shows that the learning is transferrable across environments and robust even when the auditory signal is replaced by a very obviously different signal, which, however, exhibited the same behaviour. An important parameter of training effectiveness is not only the final performance for specific tasks, but also the time it takes to achieve a given target performance. Shams and Seitz [48] argue that multisensory-training protocols can better approximate natural settings and are more effective for learning than uni-sensory training.

Fidelity evaluation forms an important part of the qualification of flight simulator training devices [12]. Most of this evaluation is currently based on subjective measures. We make a case for task specific validation that is based on objective measures of human performance as part of this qualification process. Objective fidelity measures do not substitute, but complement, subjective measures of fidelity.

Our results highlight the need for sensitive and robust performance measures and test strategies to evaluate whether individual cues contribute to overall performance. Individual cues, such as the auditory motion cue, make a statistically significant contribution to performance only when the same information is not simultaneously signalled by the motion platform. We use a sensitive test that measures performance using a factorial design within individual participants. Subtle performance differences, such as those induced by the omission of individual cues, particularly where multiple cues provide redundant information, are unlikely to be visible in performance comparisons across groups where intra-individual variability is likely to mask small effects unless very large group sizes are used (e.g. [49]). This finding may explain why for many tasks there are no measurable benefits of the use for motion platforms to transferable training (review [1]). Methodologies from basic research in multisensory perception, which provide efficient and robust paradigms for the evaluation of individual cues to perception and performance can be adapted to provide measures of objective fidelity.

## Methods

### Ethics Statement

The experiments have been approved by the University of Liverpool ethics committee (reference PSYC09100027). Written informed consent was acquired from all participants.

### Participants

Three distinct groups of 10 participants each, recruited via opportunity sampling, took part in the experiments.

Experiment 1: age range 19–45 years, mean 26; seven males.

Experiment 2: age range 19–39 years, mean 22; nine males.

Experiment 3: Range = 20–29 years, mean = 22.6, eight males.

All participants reported normal or corrected-to-normal vision and normal hearing. None had experienced the flight simulator prior to the experiments.

### Apparatus and Materials

#### The flight simulator

The HELIFLIGHT simulator [43] based at the University of Liverpool’s School of Engineering was used for testing in all experiments. The simulation was run using aircraft-specific modelling software (FLIGHTLAB), running on PC-based Linux framework.

**Figure 8:**
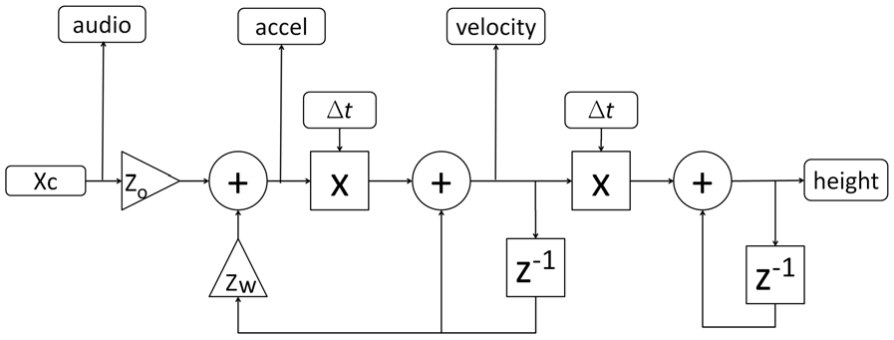
Schematic diagram of the heave (up/down) model. The control input (XC) directly controls vertical acceleration, velocity and height via the feedback coefficient Z_w._ The audio signal modulation is controlled via the control input (XC), the motion platform receives input from the first feedback loop (accel) while the visual signals directly represent the final model output (height).

The flight dynamics model used in all experiments is shown in figure 8. The model is restricted to up/down movements. The control input (Xc) could be under computer control (experiment 1) or controlled via the collective lever by the pilot (experiments 2 and 3). Two parameters govern the flight dynamics: The input gain was constant in all experiments (Z_o_ = 4.8) while the damping coefficient (Z_w_) was set to −0.1 to create difficult to control flight dynamics or to −0.5 in the ‘easy’ conditions (experiment 2 and 3). In experiment 1 Z_w_ was set to −0.1.

Visual information was presented via Optivision collimated displays, with the collimated mirrors approximately 4 feet away from the participant. The visual display consisted of a simulated flight path at 1500 ft above ground. In experiment 1 a text prompting subjects to respond to a stimulus change was displayed for 500 ms.

**Figure 9:**
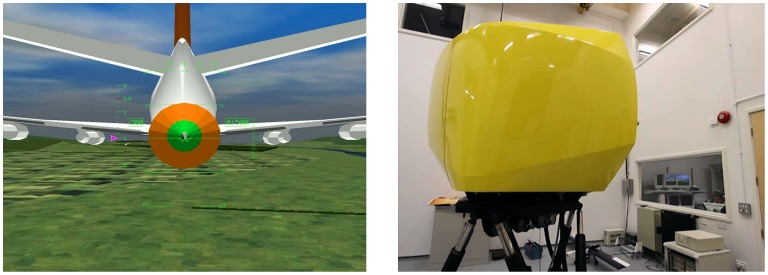
The centre visual display seen by participants (Left) in the HELIFLIGHT simulator (right). Participants were instructed to use the collective lever to control the height of the helicopter, and to keep the W-shaped gull’s wing cross-hair as close as possible to the centre of the refuelling basket.

In experiments 2 and 3 the display contained a representation of a tanker plane and refuelling basked (fig. 9).

**Figure 10:**
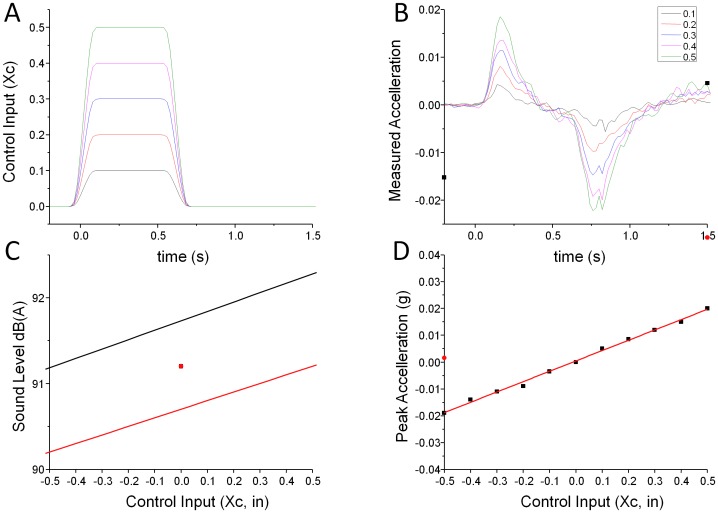
Measured motion and audio parameters in response to the control inputs used in experiment 1. The time course of the control inputs (Xc) and sample acceleration signals for these five conditions are shown in panels A and B. Straight line fits to measured audio level and peak acceleration data are shown in panels C and D. The black line in Panel C shows the level of the substitute sound (saxophone tuning note) while the red line shows amplitude in the simulated turbine condition. The symbol in the centre of the plot shows the sound level that was played during the static audio condition.

Auditory stimuli were delivered via loudspeakers in the simulator capsule at 87.5 dB(A) while the pilots wore sound attenuating headphones (Flightcom 4DLX (attenuation –24dB). The audio signal consisted of two components, the rotor sound and a turbine sound. Both sounds were continuous loops that were generated under control of a Tucker-Davies-Technologies (www.tdt.com) TDT RM1 real time processor. The turbine signal pitch and amplitude was modulated in direct proportion to the control input (fig. 10 C). The rotor playback speed (and pitch) was always constant but the rotor signal amplitude co-varied with the control input Xc (fig. 8). The overall signal level varied by 3dB (86–89 dB(A)) over the full collective range (+− 3dB). The TDT signal processor was controlled by a separate computer via a network connection and modulated the auditory signals in real time, delays due to communication lags were below 20 ms. The auditory signal was designed such that changes were easily audible: Experiment 1 shows that the detection threshold for the audio signal component for untrained participants was a control input of 0.42 in, the *average* absolute control input during our experiment 2, where novice pilots were in control of the simulation was 0.91 in.

Kinematic cues were delivered via a Maxcue 600 series motion platform. Platform motion was restricted to vertical movements, which were controlled by computer (experiment 1) or under the control of the participants (experiment 2 and 3). The acceleration signal (accel in fig. 8) was used to drive the motion platform. A washout filter was used to deliver realistic motion cues within the restricted simulator workspace [50]. Figure 10 C, shows measured acceleration data in response to the control input modulations used in experiment 1 (fig 10 A). The peak acceleration is well described by a slope of 0.04 g/Xc (fig 10 D). The kinematic motion signal is delayed by approximately 80 ms relative to the onset of the control input.

Other features of the capsule included a realistic helicopter control set-up, including a collective lever to the left of the pilot’s seat which was be used for vertical movement of the ‘helicopter’ in Experiments 2 and 3 and a cyclic control with a top-hat button that was used by participants to respond in experiment 1. The instrumentation panel was off during all experiments.

#### The simsim

**Figure 11:**
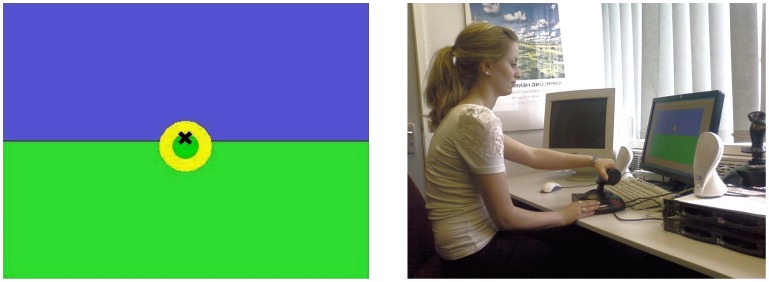
The ‘simulation simulation’ (simsim). The visual display (left) captures only the essentials of the full simulation but both simulations share an identical flight dynamics model and audio signals. Participants use a throttle lever (right) to position the cross inside the circle. The simulation did not provide kinematic cues.

A reduced fidelity ‘simulator simulator’ (simsim) was used in experiment three to train participants to exploit auditory cues. The simsim had a flight dynamics model and audio representation that was identical to the high fidelity simulator, but did not have a motion platform. Visuals were provided on a single 17″ LCD screen and consisted of a vertically moving yellow circle representing the refuelling basket height and a black cross that had to be aligned with the circle, fig. 11.

A throttle controller, part of a commercial joystick (Thrustmaster T-Flight Hotas X), was used to control the input to the heave model. All subjects used their left hand as in the full simulation.

**Figure 12:**
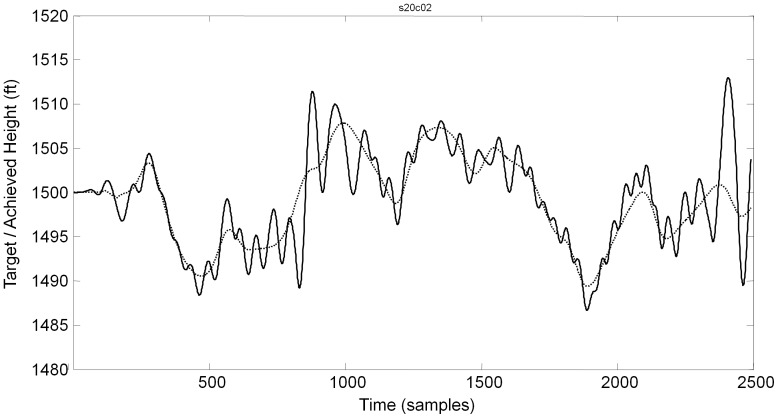
Sample flightpath (continuous line) and basket motion (dotted line) for a difficult flying condition. This participant oscillates around the target position. The median absolute distance between actual flying height and the target position is used as an error measure.

The target moved at a predetermined path which participants were required to follow for 15 minute training blocks. The height variation was defined as a sum of 6 sinusoidal signals, with frequencies ranging from 0.2 to 0.5 rad/s, each with a different amplitude and phase shift, giving the path shown in figure 12.

Sound was always played (at around 66 dB(A)) via JBL duet (www.jbl.com) loudspeakers. All other sound parameters were identical to those used in the full simulator. Participants were instructed to follow the target as closely as possible.

#### Performance evaluation

In experiment 1 participants were asked to report the direction (up/down) of changes to the auditory signal, kinematic (motion platform) motion or both. A visual cue in the centre of the visual display coincided with a signal change and subjects were required to indicate whether the direction of motion was up or down by moving a button on the simulator cyclic stick up or down. Subjects were instructed to respond even if no change was perceived. Mean correct response rates were computed for a pseudorandom sequence of control input variations corresponding to collective displacements of 0.1, 0.2, 0.3, 0.4, 0.5 inches, fig. 10. Each of the 30 conditions (10 levels of auditory, kinematic and auditory plus kinematic signal modulation) was reported. The total experiment took less than 30 minutes for each of ten untrained participants to complete. Trials where no response or more than one response were given are discounted.

The behavioural task in experiments 2 and 3 was to follow the path of a refuelling basket attached to the tail of a plane (fig. 9). The distance between the crosshair on the visual display and the centre of the target indicated how closely the target was followed. The vertical trajectory of the basket was predetermined by the computer (there was no horizontal movement). Flightpaths for each test point, whilst identical, were designed to be too complex for participants to learn (fig. 12). A randomised block design was used for experiments 2 and 3 to control for learning effects.

Subject performance was quantified as the median absolute difference between the target and the actual height. Figure 12 shows a sample flight path (dotted line) and the flown trajectory.

Learning progress in experiment 3 was monitored for all participants by evaluating linear fits to the mean tracking error over the four training intervals and the two task difficulty settings. To ensure that only participants that showed improvements during the training session are included we defined a minimum improvement of 0.15 ft mean error to be included in the analysis.
